# Potential Habitat and Priority Conservation Areas for Endangered Species in South Korea

**DOI:** 10.3390/ani15081158

**Published:** 2025-04-17

**Authors:** Soyeon Park, Hyomin Park, Sangdon Lee

**Affiliations:** 1 Department of Environmental Science & Engineering, Ewha Womans University, Seoul 03760, Republic of Korea; psy25730@ewha.ac.kr (S.P.); hyomin@ewha.ac.kr (H.P.); 2 Carbon Neutrality Support Center, The Hwaseong Institute, Hwaseong-si 18469, Republic of Korea

**Keywords:** MaxEnt, Zonation, priority area, gap analysis, conservation strategy

## Abstract

Anthropogenic threats from human activity have significantly altered global ecosystems, resulting in habitat destruction and fragmentation, biodiversity loss, and species extinction. The identification and establishment of protected areas for the conservation of threatened species is thus required to mitigate the impacts of these threats. This study identified priority conservation areas (PCAs) for endangered mammals, birds, amphibians, and reptiles in South Korea using ecological modeling. In particular, we used the MaxEnt model to determine the potential distributions of endangered species and Zonation software to prioritize conservation areas. In doing so, we aimed to identify key environmental factors that influence the distributions of the target species and to identify ecologically critical areas using gap analysis based on the first-grade areas on the ecology and nature map (ENM). The results indicated that the highest priority areas for endangered species varied by taxon, with endangered mammals found primarily in forested areas, endangered birds on inland plains, and endangered amphibians and reptiles within marine and stream regions. The gap analysis revealed a very low overlap between ecological areas and high-priority areas for endangered birds and amphibians. This suggests that the first-grade areas on the ENM mainly represent areas with high vegetational coverage. These findings highlight the need to reassess existing protected areas and designate PCAs for endangered species independently from the ENM. This study represents a foundational assessment that can be used to inform conservation planning and prompt continued ecological research in support of biodiversity conservation efforts.

## Simple Summary

Identifying priority conservation areas is crucial for effectively protecting endangered species. Using species distribution modeling and priority analysis for endangered mammals, birds, amphibians, and reptiles in South Korea, we found that the environmental factors influencing habitat selection and the spatial patterns of priority areas differ across taxonomic groups. In addition, we identified high-priority conservation areas that overlap with ecologically valuable regions, emphasizing their importance for biodiversity preservation. Our results suggest the need for further ecological research on endangered birds, amphibians, and reptiles, which remain relatively understudied. Overall, this study underscores the importance of priority analysis in the designation of protected areas and habitat conservation strategies considering the ecology of endangered species.

## 1. Introduction

Biological diversity is a key driver of ecosystem functions that arise from the interactions between species and their environments [1]. However, industrialization and associated human activity have significantly altered global ecosystems, accelerating biodiversity loss and species extinction [2]. In particular, habitat loss and fragmentation caused by industrial development are major threats to biodiversity because they prevent species from finding suitable environments [3,4,5]. These environmental changes, combined with the failure of many species to adapt, have increased the risk of extinction for many taxa and have led to a major loss of global biodiversity. Effective conservation strategies are thus required to mitigate this loss of biodiversity driven by anthropogenic threats. Effective conservation strategies require a systematic understanding of the ecology and current status of the target species [6]. As a result, there has been strong global interest in investigating the environmental factors that affect the distribution of wildlife and in compiling relevant data to halt the decline in biodiversity. For example, the International Union for Conservation of Nature (IUCN) has compiled a comprehensive list of endangered species and their distribution, habitat conditions, and main threats in the IUCN Red List, which is a key resource for biodiversity conservation research [7,8].

Information on endangered species is essential for devising effective strategies for biodiversity conservation, but collecting data on these species is hindered by their small population sizes and limited habitats. As a result, there has been a recent focus on determining the current status and potential distribution of endangered species using modeling techniques. For example, species distribution models (SDMs) are widely used to predict species distributions based on species data and environmental variables [9]. Common SDMs include the generalized linear model (GLM), the genetic algorithm for rule-set production (GARP), and maximum entropy (MaxEnt), which have been employed to predict potential habitats [10], evaluate habitat quality [11], and identify critical habitats [12]. Various studies have also employed SDMs for endangered species protection in South Korea, but their focus has primarily been on analyses of single species [13]. Given the increasing number of endangered species, there is a growing need to develop conservation strategies that consider biodiversity and ecosystem preservation for multiple endangered species rather than focusing solely on individual species [14]. Therefore, further research is required to establish multi-species conservation strategies tailored to the ecological conditions of South Korea.

One of the most effective strategies for biodiversity conservation is the designation of protected areas (PAs). PAs require a sufficient range covering the ecological regions and habitats of key species to maximize conservation outcomes [15,16,17]. Identifying areas with a high probability of containing endangered species can be used to assess ecosystem health and provide essential data for the establishment of PAs. Therefore, research is needed to predict the potential habitat of endangered species using SDMs and prioritize conservation areas based on the results. Mapping priority conservation areas (PCAs) within species habitats facilitates the development of systematic conservation plans, a process supported by quantitative tools such as Zonation (version 2.0), Marxan, and C-Plan [18]. During this process, it is also important to verify that current PAs adequately cover the habitat of endangered species. In this context, gap analysis can be used to identify high-biodiversity areas that are underrepresented within existing PA networks, thus guiding the designation of new conservation areas [19].

Few previous studies share a similar methodology to prioritize habitats of endangered species in South Korea. For example, Mo (2018) conducted priority conservation analysis using Marxan for endangered mammals, birds, amphibians, reptiles, and fish and set different conservation purposes for Endangered Wildlife Class I and Class II [20]. Jeong et al. (2024) utilized the MaxEnt model and InVEST Habitat Quality model for predicting and assessing the habitat status of two endangered mammals, conducting gap analysis with existing PAs in South Korea to derive PCAs [21]. Other studies have primarily focused on a single species or one taxonomic group. Therefore, this study using a priority analysis of multiple endangered species can serve as another basis for designating PAs and supplementing the conservation strategies.

The present study was conducted in South Korea to analyze the potential habitats for endangered mammals, birds, amphibians, and reptiles and the environmental factors affecting species distributions using the MaxEnt model. Based on the results, PCAs for each taxonomic group were then identified using Zonation software. Finally, core habitat areas for endangered species were determined using gap analysis by overlapping high-priority areas (HPAs) with the first-grade areas on the ecology and nature map (ENM). This study thus aimed to provide guidelines for the development of effective conservation strategies for the protection of endangered species in South Korea by identifying PCAs and providing a basis for setting priorities during the decision-making process when establishing PAs.

## 2. Materials and Methods

### 2.1. Study Site

**Figure 1:**
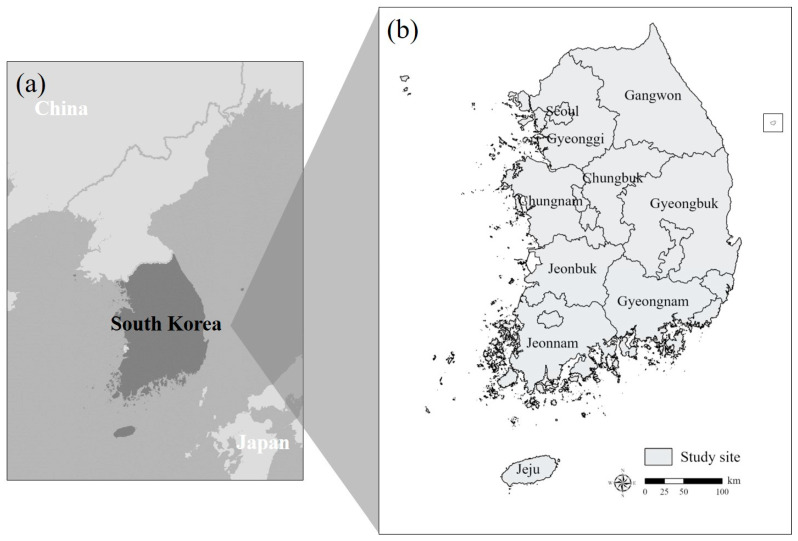
(**a**) Location of the study site, situated between China in the west and Japan in the east, and (**b**) provincial areas in the Republic of Korea.

The present study was conducted in South Korea, a country in East Asia located on the Korean Peninsula, which extends from 33° to 44° latitude and from 124° to 132° longitude (Figure 1a). In terms of climate, South Korea has four distinct seasons because it is situated in the mid-latitude temperature climate zone [22]. The average annual temperature is approximately 13.2 °C, with an average annual precipitation of 1237.4 mm [23]. The total study area covers approximately 100,413 km^2^ [24]. About 70% of the study area consists of forested terrain and, although the elevation is relatively low compared to other countries, the region has a complex topographic structure [25]. The geographical distribution of endangered species was investigated across nine administrative regions: Gyeonggi-do (Gyeonggi), Gangwon-do (Gangwon), Chungcheongbuk-do (Chungbuk), Chungcheongnam-do (Chungnam), Gyeongsanbuk-do (Gyeongbuk), Gyeongsangnam-do (Gyeongnam), Jeonbuk-do (Jeonbuk), Jeollanam-do (Jeonnam), and Jeju-do (Jeju) (Figure 1b).

### 2.2. Study Species

Currently, 282 species are designated as endangered in South Korea, including 20 mammal species, 69 bird species, 4 amphibian species, 4 reptile species, 29 fish species, 29 insect species, 32 invertebrate species, 92 terrestrial plant species, 2 marine alga species, and 1 higher fungus species [26]. We selected target species from the list of mammals, birds, amphibians, and reptiles designated as Endangered Wildlife Class I or Class II by the Ministry of Environment (MOE), with particular consideration given to species that have already been the subjects of major research in South Korea. Only those species that have had their occurrence confirmed by field surveys (e.g., via track records) were included, while species that have already become extinct in South Korea, such as *Panthera tigris altaica*, *Canis lupus coreanus*, and *Lynx lynx*, and species that have only been documented based on interviews or literature reviews were not considered. Using insufficient species occurrence data for training an SDM increases the risk of model overfitting, leading to overly optimistic predictions that distort the actual distribution patterns, thereby reducing the reliability of the model outcomes [27]. For reliable SDM outcomes, a sufficient number of occurrence records are required [28], and at least 15 records are necessary for statistical analysis [17]. Therefore, we selected endangered species with at least 15 occurrence records based on the Nationwide Natural Environmental Survey (NNES) conducted by the National Institute of Ecology (NIE). This resulted in a total of 15 endangered wildlife species being selected for this study: five mammal species (leopard cat (*Prionailurus bengalensis*), Eurasian otter (*Lutra lutra*), yellow-throated marten (*Martes flavigula*), Siberian flying squirrel (*Pteromys volans*), long-tailed goral (*Naemorhedus caudatus*)), four bird species (Eurasian hobby (*Falco subbuteo*), Chinese sparrowhawk (*Accipiter soloensis*), Eurasian sparrowhawk (*Accipiter nisus*), northern goshawk (*Accipiter gentilis*)), three amphibian species (narrow-mouthed toad (*Kaloula borealis*), Korean golden frog (*Pelophylax chosenicus*), Suweon tree frog (*Dryophytes suweonensis*)), and three reptile species (rat snake (*Elaphe schrenckii*), Korean tiger lizard (*Eremias argus*), Reeve’s turtle (*Mauremys reevesii*)) (Table A1).

### 2.3. Species Distribution Prediction

#### 2.3.1. MaxEnt Model

Due to the limited availability of occurrence data for endangered species, this study employed the MaxEnt model because it allows for analysis based solely on species occurrence records. The MaxEnt model can predict the probability of species distribution based on species occurrence data and environmental variables associated with the preferred habitat of the target species. Because this model generates indicators of habitat suitability, it has been widely used in past ecological modeling research [29,30]. The MaxEnt model runs using species occurrence data as the dependent variable and environmental factors as continuous or categorical independent variables, outputting the species distribution probability based on each environmental variable. The model’s performance can be validated using the area under the receiver operating characteristic (ROC) curve. An area under the curve (AUC) closer to 1 indicates high predictive accuracy. Generally, model results with an AUC of 0.7 or higher are considered reliable [31].

We used MaxEnt (version 3.4.4) with logistic regression and bootstrapping. In this process, 70% of the occurrence data were randomly allocated as training data, and the remaining 30% were used as test data for model validation. The MaxEnt model incorporates regularization to improve model performance by mitigating model overfitting. The model also provides five feature types—linear (L), quadratic (Q), product (P), threshold (T), and hinge (H)—that reduce overfitting by allowing for a flexible representation of the relationships between species occurrence and environmental variables [31]. When feature types are left at their default settings, the model automatically selects features depending on the number of occurrence records, resulting in inconsistencies across taxonomic groups. Therefore, we used all five feature types to capture linear and non-linear relationships between species occurrence and environmental variables [32]. Notably, presence-only data are primarily collected from accessible areas, which can lead to sampling bias in model outcomes [33]. To address this problem, adjusting model parameters such as sample bias can be used to prevent spatial bias in species distribution [10,34]. In this study, we applied taxon-specific bias files to minimize spatial sampling bias. The model was run 10 times for each taxonomic group, and the jackknife test was used to determine the contribution to model predictions with response curves. The ROC curve was used to evaluate the predictive performance of the model results by considering sensitivity and specificity. A higher sensitivity and a lower false positive rate (i.e., higher specificity) indicate a good model performance. The optimal threshold for distinguishing species presence and absence is determined at the point where the sum of sensitivity and specificity is maximized [35]. Therefore, we used the maximum training sensitivity plus specificity (MTSS) threshold used to classify the predicted habitats into either present (1) or absent (0) to determine habitat suitability. The derived maps showing the suitable habitat for each taxon were then used as input for the selection of PCAs using Zonation software.

#### 2.3.2. Species Occurrence Data

Species occurrence data for the MaxEnt model were obtained from the 4th NNES conducted by the NIE. The NNES assesses the status of ecosystems and biodiversity across South Korea using field surveys collecting data on topography, vegetation naturalness, habitat conditions for endangered wildlife, and biodiversity [36]. The 4th NNES was conducted over a five-year period from 2014 to 2018 in order to enhance the accuracy of ENM data. In this version of the NNES, the data were collected at a landscape scale by dividing a 1:25,000 topographic map into nine grid units, with nine categories assessed: topography, vegetation, flora, benthic macroinvertebrates, terrestrial insects, fish, amphibians and reptiles, birds, and mammals [36].

The 4th NNES dataset included 7794 records for mammals, 1963 for birds, and 352 for amphibians and reptiles. In the present study, we excluded data with notation errors for location information and missing data. We then used the spatial thinning method in the spThin package in R studio (version 4.4.3) to remove spatial bias by distributing species occurrence data at 1 km intervals. After preprocessing, a total of 6166 occurrence points for endangered species were used as input data for the MaxEnt model, including 4437 points for mammals (*P. bengalensis*: 2374; *L. lutra*: 1481; *M. flavigula*: 446; *P. volans*: 85; *N. caudatus*: 51), 1452 points for birds (*F. subbuteo*: 488; *A. soloensis*: 466; *A. nisus*: 284; *A. gentilis*: 214), 174 points for amphibians (*K. borealis*: 88; *P. chosenicus*: 60; *D. suweonensis*: 26), and 103 points for reptiles (*E. schrenckii*: 63; *E. argus*: 25; *M. reevesii*: 15) (Table 1).

#### 2.3.3. Environmental Variables

The environmental variables used for the MaxEnt model were selected based on key factors employed in previous studies. These were classified into topographic (elevation, slope, aspect, and topographic wetness), distance-related (distance from a residential area, water, a used area, an agricultural area, or a road), land cover, climate, and vegetation (vegetation index and forest age class) variables [17,37,38,39,40,41,42].

Elevation data were obtained from the digital elevation model provided by the National Geographic Information Institute (NGII) of the Ministry of Land, Infrastructure and Transport (MOLIT) (https://map.ngii.go.kr, accessed 11 October 2023). Based on this dataset, slope and aspect variables were generated through spatial analysis using ArcGIS. Land cover data were sourced from a 2023 mid-class land cover map (5 m × 5 m resolution) provided by the Environmental Geographic Information Service (EGIS) of the MOE (https://egis.me.go.kr, accessed 9 May 2024). The five distance variables were extracted based on land cover classification codes. For example, in the case of residential areas, data were derived based on land cover classification code (110), defined as residential area specified in EGIS. Every distance variable was created using the Euclidean distance tool in ArcGIS. The topographic wetness index (TWI) and the normalized difference vegetation index (NDVI) were obtained from the environmental big data platform provided by the Korea Institute of Geoscience and Mineral Resources (KIGAM) (https://www.bigdata-environment.kr, accessed 20 June 2024). Climate variables were derived from 19 bioclimatic variables from WorldClim (https://worldclim.org, accessed 20 June 2024). For the MaxEnt model input data, two temperature-related variables (Bio3 and Bio4) and two precipitation-related variables (Bio16 and Bio17) were selected based on correlation analysis. Vegetation data were obtained from a 1:5000 forest type map provided by the Forest Geospatial Information System (FGIS) of the Korea Forest Service (KFS) (https://map.forest.go.kr, accessed 21 September 2023). Based on forest type map codes, the forest type, forest age class, forest diameter class, and forest density were extracted. Highly correlated data were then excluded using correlation analysis, leading to the use of only forest age class as input data (Table A2).

**Figure A1:**
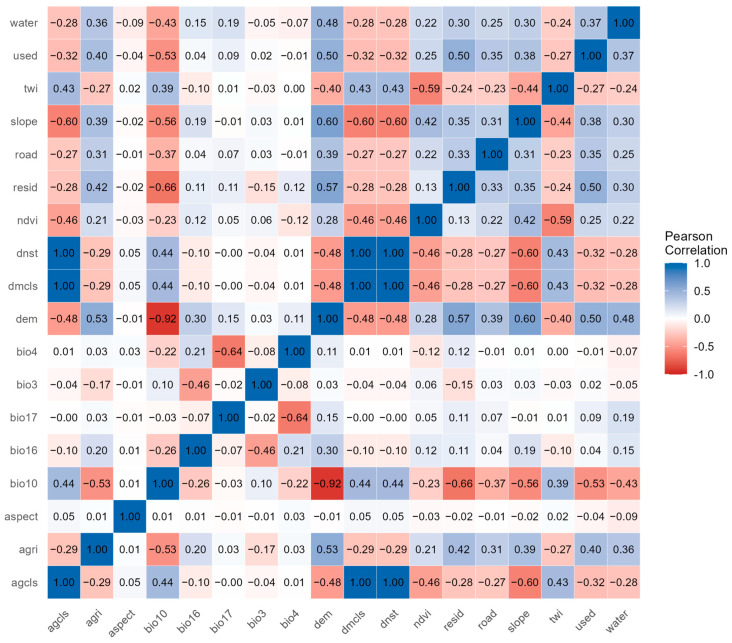
Results of Pearson’s correlation analysis for the environmental variables.

All environmental variables were generated at a 1 km × 1 km resolution in the EPSG: 5186 coordinate system using ArcGIS Pro (version 3.1.3). To avoid multicollinearity, Pearson’s correlation analysis was conducted using ENMTools in R studio (version 4.4.3), and variables with a high correlation coefficient (|r| > 0.7) were excluded [43,44] (Figure A1). In total, 16 environmental variables were selected as MaxEnt model inputs (Table A3).

#### 2.3.4. Spatial Prioritization

Zonation is a decision-supporting software that spatially prioritizes areas by utilizing spatial data for biodiversity features associated with species, habitats, and ecosystem services [45]. Zonation priority analysis operates under the Zonation meta-algorithm, which generates an iterative cell removal rule based on marginal loss, a metric that quantifies the reduction in overall biodiversity value resulting from the removal of individual cells [46,47]. The conservation value of each cell is evaluated by its spatial distribution and ecological importance, and cells with the lowest priority are sequentially removed following one of the principal rules: additive benefit function (ABF) and core area zonation (CAZ). ABF supports the overall maintenance of biodiversity, whereas CAZ emphasizes the protection of core habitats critical for rare or endangered species [48]. Zonation allows for flexible use in various types of inputs based on user-specific objectives. For example, hierarchic analysis assigns a priority value of 1 to hierarchic mask layers representing PAs and a value of 0 to other regions, ensuring that HPAs are excluded from the cell removal rule [49]. The hierarchic mask layer can effectively safeguard designated conservation areas by preventing their exclusion during the prioritization process. Based on the type and attributes of the inputs, Zonation estimates the biodiversity value of spatial units and ranks the priority of each unit from lowest (0) to highest (1) in terms of their importance for conservation [50]. Zonation generates three main outputs for priority analysis results: priority rank maps, performance curves, and histograms. The priority rank maps visualize the value of the landscape based on the conservation priority for each spatial unit, while the performance curves indicate the distribution of priority rankings based on specific features of the input data and the histograms summarize the distribution of input data features in areas rated as the highest priority. Unlike SDMs, Zonation is not capable of statistical analysis to assess the interrelationships between variables. Instead, it employs the output of other models as input data. For example, the results of SDMs that include spatial information for each cell are often directly used as input data for various prioritization techniques, including Zonation [49]. The analysis of habitat quality and ecosystem connectivity can also be conducted based on costs and threats; thus, Zonation can serve as a quantitative methodology for the sustainable management of biodiversity [49].

The present study employed the MaxEnt results as input data for Zonation 5 (version 2.0) in order to prioritize conservation areas within potential habitats that were common to all taxonomic groups. The input data used to prioritize potential habitats in Zonation were the average values taken from the species-specific distribution predictions produced by MaxEnt, with equal weighting applied [6]. We also used a hierarchical mask layer to determine whether an area should be evaluated based on the habitat use [51]. Therefore, this study created binary maps based on MTSS thresholds from the MaxEnt results distinguished by species presence (1) and absence (0) to identify common suitable areas (SAs) for each taxon. These binary maps thus allocated priority scores according to habitat suitability for each taxonomic group. All input data used for the analysis were generated at the same resolution, coordinate system, and spatial extent in ArcGIS pro, consistent with the preprocessing conducted for the MaxEnt modeling. The datasets were subsequently converted to TIFF format for use in the Zonation analysis. We applied the CAZ rule for priority analysis, and aside from the implementation of hierarchical analysis, all other parameters were maintained at Zonation default settings [48,52]. Spatial analysis was then conducted with the Zonation results using natural breaks to divide the regions into five categories, confirming the PCAs within the potential habitats [53]. These PCAs were selected based on regions with the highest priority rank (i.e., HPAs).

#### 2.3.5. Gap Analysis

Gap analysis is widely used to assess gaps between newly proposed conservation areas and existing PAs by geographically overlapping species distribution data with environmental datasets [19,21,54]. In this study, we conducted gap analysis using MaxEnt-derived habitat suitability maps for the target endangered species and the ENM, which evaluates ecological conservation areas in South Korea.

The ENM is a 1:25,000 scale map of South Korea constructed based on the results of 14 national ecosystem surveys. It classifies natural environments such as mountains, rivers, and inland wetlands by region according to their ecological value, naturalness, and landscape significance [55]. The four main factors that determine the ENM classification are vegetation, endangered wildlife, wetlands, and topography. The ENM categorizes regions into 1st-grade areas, within which the conservation and restoration of the natural environment are prioritized; 2nd-grade areas, which serve as buffer zones and are subject to restricted use; and 3rd-grade areas, within which development is permitted. Separate management areas are also designated for specific protection purposes [55]. The 1st-grade areas identified on the ENM are important for ecological conservation and require strict protection and restoration efforts. They are considered habitats for endangered wildlife (Classes I or II) or contain high-value vegetation (Vegetation Conservation Grades I or II) [45]. While the ENM serves as a vital indicator for evaluating ecosystem conservation and environmental impact assessment in development planning, the proportion of 1st-grade land remains relatively low compared to its ecological significance [56].

**Figure 2:**
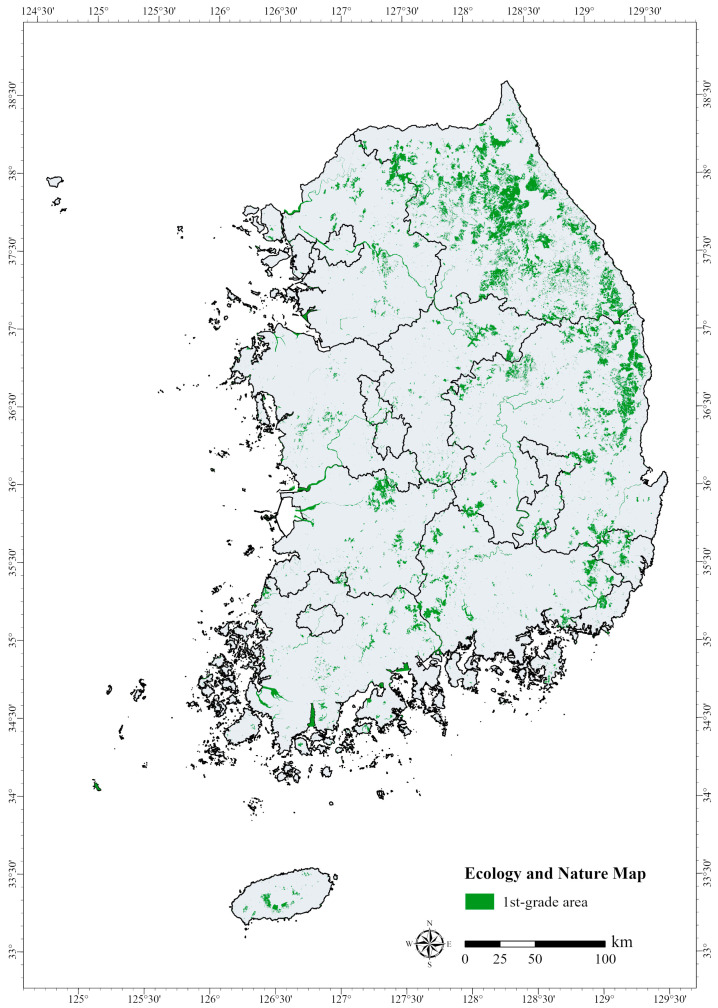
Ecology and nature map showing the 1st-grade areas.

Therefore, this study used gap analysis to examine core PCAs (CPCAs) based on the spatial overlap between the Zonation-derived HPAs and the 1st-grade areas on the ENM. We aimed to identify high-probability occurrence areas for endangered species that remain unprotected by government-designated PAs, highlighting regions where urgent conservation strategies are required (Figure 2). For this analysis, 2023 data from the ENM were obtained using the EGIS of the MOE (https://egis.me.go.kr, accessed 9 September 2024). The 1st-grade areas were extracted using ArcGIS Pro (version 3.1.3) and converted into a raster format for comparison with HPAs for endangered species.

## 3. Results and Discussion

### 3.1. Distribution of Endangered Mammal Species

This study used the MaxEnt model to predict the distribution of five endangered mammals: *P. bengalensis*, *L. lutra*, *M. flavigula*, *P. volans*, and *N. caudatus*. The AUC was 0.658 for *P. bengalensis*, 0.743 for *L. lutra*, 0.823 for *M. flavigula*, 0.793 for *P. volans*, and 0.964 for *N. caudatus* (Table 2). An AUC value of 0.7 or higher is generally considered to indicate an adequate fit for model predictions [31,57]. Based on this criterion, the model results for *L. lutra*, *M. flavigula*, *P. volans*, and *N. caudatus* demonstrated high predictive performance. However, the AUC for *P. bengalensis* was lower than that for the other mammal species. This was in accordance with previous studies that have reported AUC values ranging from 0.6 to 0.7 for *P. bengalensis* [37,38,58]. The relatively low AUC for *P. bengalensis* can be attributed to its broad ecological plasticity, inhabiting forests, wetlands, plains, and agricultural areas, while the other target mammal species have lower ecological plasticity [59].

The environmental variables influencing the distribution of the five endangered mammals and their contribution to the model outputs were also analyzed (Table 2). For *M. flavigula* and *P. volans*, elevation (DEM) had the highest contribution (42.9% and 18.7%, respectively), suggesting that the likelihood of occurrence for both species increased at higher elevations. This is consistent with previous studies on the ecology of *M. flavigula* [60,61] and *P. volans* [62,63] that have reported that both species predominantly inhabit forested areas within the Baekdudaegan Mountain Range, which stretches across the Korean Peninsula. For *L. lutra*, the distance from water (Water) was the most important variable (33.8%), which was also in accordance with the results of previous studies [64]. It is known that *L. lutra* has a strong dependence on riverine habitats such as streams and rivers [65]. For *P. bengalensis* and *N. caudatus*, the model results revealed that the highest contributions came from climatic variables, in particular temperature seasonality (Bio4) for *P. bengalensis* and precipitation in the driest quarter (Bio17) for *N. caudatus*. Previous studies on *P. bengalensis* have found that temperature-related variables had the strongest predictive effect on their distribution [66]. For *N. caudatus*, the results suggest that climatic factors may be linked to their habitat selection, which is in line with previous studies on the effect of climate change on their distribution [67,68].

**Figure 3:**
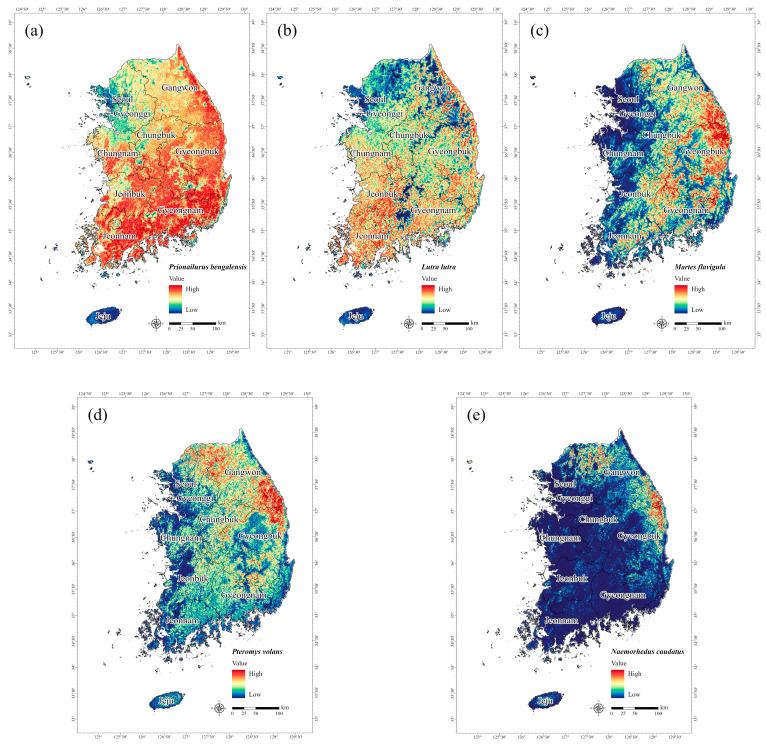
Potential distribution of five mammals designated as endangered in South Korea: (**a**) *Prionailurus bengalensis*, (**b**) *Lutra lutra*, (**c**) *Martes flavigula*, (**d**) *Pteromys volans*, and (**e**) *Naemorhedus caudatus*. Red shading represents a high probability of species occurrence.

The species distribution maps generated by the MaxEnt model represent the habitat suitability of target species with values ranging from 0 to 1, with areas shaded in red corresponding to a high probability of species occurrence. To investigate the distribution of endangered mammals based on their preferred habitat types, we analyzed potential habitats using the species distribution prediction maps. The results revealed that *P. bengalensis* exhibited the most widespread distribution among the five mammals (Figure 3a). Previous studies have also reported that *P. bengalensis* is highly adaptable to various environments, including forested areas [69]. For *L. lutra*, the map indicated a high probability of occurrence in aquatic environments (Figure 3b), which was due to their linear habitat preference centered around rivers, as documented in previous studies [70,71,72]. The maps for *M. flavigula*, *P. volans*, and *N. caudatus* highlighted a high probability of occurrence in densely forested areas from the eastern to southern regions of the Korean Peninsula and along the Baekdudaegan Mountain Range. Of these three species, *M. flavigula* had the widest distribution across Gangwon, Gyeongbuk, and Gyeongnam because this species generally has a wider home range than other medium-sized and large mammal species [60]. *P. volans*, which glides between trees for movement [73], was predicted to be found in forested areas (Figure 3d), while the potential distribution of *N. caudatus* was primarily located in Gangwon and northern Gyeongbuk (Figure 3e), reflecting the ecological characteristics of the species, being found in small populations in high-altitude mountainous areas [74].

**Figure A2:**
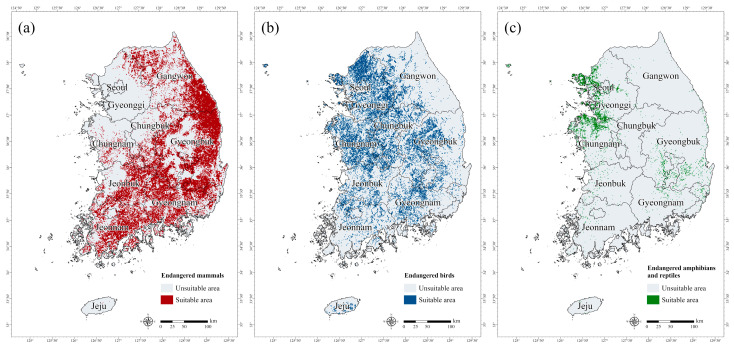
Suitable habitat area for the (**a**) five endangered mammals, (**b**) four endangered birds, and (**c**) six endangered amphibians and reptiles.

The species-specific results from the MaxEnt model were converted into binary maps using the MTSS threshold, classified according to presence (1) and absence (0). Using these binary maps, we identified the common SAs for the five endangered mammals (Figure A2a). As a result, the SAs for the endangered mammals together covered approximately 34,115 km^2^, accounting for 34% of South Korea’s total land area (100,413 km^2^). The SAs were primarily located in Gangwon, Gyeongbuk, and parts of Jeonbuk. This distribution likely reflected the habitat preferences of four of the five target species—*P. bengalensis*, *M. flavigula*, *P. volans*, and *N. caudatus*—which predominantly inhabit forested environments. Consequently, the SAs for endangered mammals were concentrated in Gangwon and Gyeongbuk, which are characterized by the presence of mountain ranges. In particular, the wide potential distribution of *P. bengalensis*, the most broadly distributed species of the five target mammals, likely contributed to this pattern.

**Figure 4:**
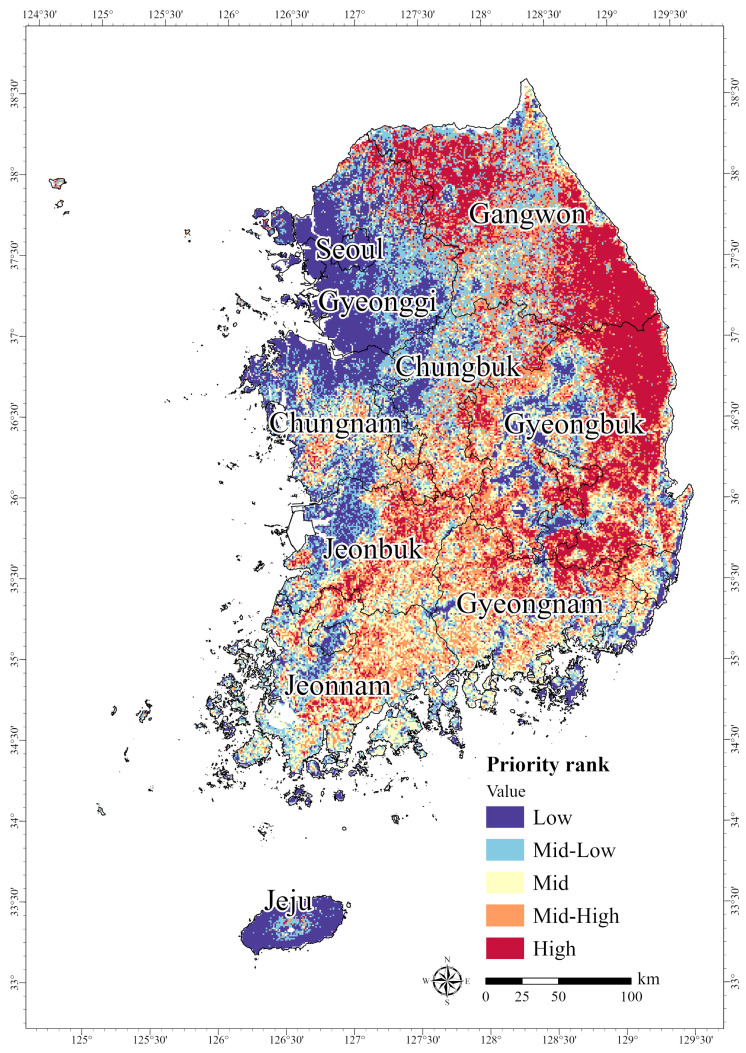
Prioritized areas of potential habitat for five mammals designated as endangered in South Korea: *Prionailurus bengalensis*, *Lutra lutra*, *Martes flavigula*, *Pteromys volans*, and *Naemorhedus caudatus*.

Analysis of the priority rankings derived from the MaxEnt model and Zonation indicated that HPAs for endangered mammal habitats were primarily located in high-altitude forests and forest edges along the Baekdudaegan Mountain Range, particularly in Gangwon and Gyeongbuk (Figure 4). Based on these results, we examined the distribution of priority ranks across the nine administrative regions. The HPAs for endangered mammals (Grade 5, “High”) covered 7218 km^2^ in Gangwon, followed by Gyeongbuk with 6973 km^2^. For all of the study sites combined, Grade 1 conservation areas (“Low”) covered 18,821 km^2^; Grade 2 (“Mid-Low”) and Grade 3 (“Mid”) together covered 19,211 km^2^; Grade 4 (“Mid-High”) spanned 19,214 km^2^; and Grade 5 (“High”) accounted for 19,590 km^2^ (Table 3). Therefore, conservation efforts should prioritize regions in Gangwon and Gyeongbuk, which have HPAs that include the habitats of the five endangered mammals.

### 3.2. Distribution of Endangered Bird Species

This study used the MaxEnt model to predict the distribution of four endangered birds: *F. subbuteo*, *A. soloensis*, *A. nisus*, and *A. gentilis*. The AUC was 0.725 for *F. subbuteo*, 0.755 for *A. soloensis*, 0.716 for *A. nisus*, and 0.738 for *A. gentilis* (Table 4). The AUC values for these birds were generally lower than those for the target mammals due to their higher mobility, making it more difficult to accurately delineate their habitat [75].

An analysis of the environmental variables influencing the potential distribution of each bird species revealed that elevation (DEM) contributed the most to the *F. subbuteo* (41.8%) and *A. soloensis* (14.9%) distributions (Table 4). The high contribution of elevation was in accordance with previous studies indicating that the probability of occurrence for these species tends to decrease at higher altitudes [76,77]. For *A. nisus*, which is known to prefer riverine environments [26], distance from water (Water) was the most influential variable, contributing 11.7% to the results. For *A. gentilis*, temperature seasonality (Bio4) had the strongest impact at 21.5%, which is consistent with previous research showing that climatic variables significantly influence this species’ habitat selection during the breeding season [78].

**Figure 5:**
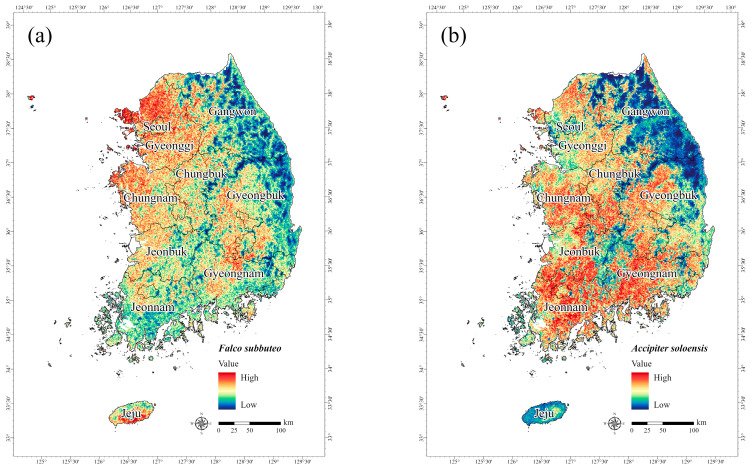

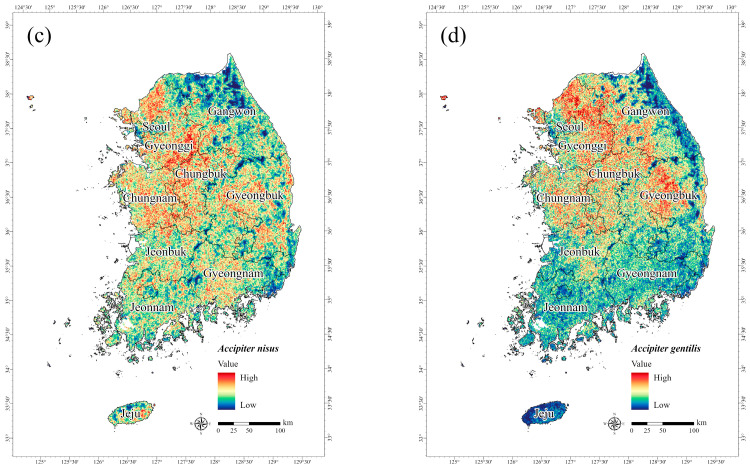
Potential distribution of four birds designated as endangered in South Korea: (**a**) *Falco subbuteo*, (**b**) *Accipiter soloensis*, (**c**) *Accipiter nisus*, and (**d**) *Accipiter gentilis*. Red shading represents a high probability of species occurrence.

The potential habitats of the endangered birds generated by the MaxEnt model were visualized as species distribution maps, with areas in red indicating a high probability of species occurrence. Unlike the endangered mammals, the four endangered birds had a lower likelihood of occurrence in forested areas in Figure 5, but as birds differ in environment preference during breeding and non-breeding seasons, this is not a complete reflection of their habitat use. For *F. subbuteo*, the results revealed a high probability of occurrence, primarily in Gyeonggi (Figure 5a). *F. subbuteo* is known to be distributed throughout South Korea during its breeding season from May to August, with a preference for plains, wetlands, and agricultural areas for foraging [26]. Therefore, the frequency of occurrence was high in Gyeonggi, where the relevant environment is developed [76]. Of the four target bird species, *A. soloensis* had the widest distribution on the map, with a high probability of occurrence in Chungnam, Jeonbuk, Jeonnam, and Gyeongnam (Figure 5b). Past research has reported that *A. soloensis* has a nationwide distribution centered on forests, plains, and agricultural areas [76]. The present study also revealed a low probability of *A. soloensis* occurring in high-altitude areas (Table 4); thus, the probability of *A. soloensis* inhabiting the parts of Gyeongbuk and Gangwon with mountainous regions was correspondingly low. This finding is consistent with previous research indicating that *A. soloensis* is frequently observed in most areas except for high mountains in Gangwon and urban areas in some inland regions [76]. *A. nisus* and *A. gentilis*, both of which are wintering species in South Korea, are known to inhabit forests and agricultural areas across the country [26]. In the present study, the prediction results for *A. nisus* (Figure 5c) and *A. gentilis* (Figure 5d) showed a higher probability of occurrence in inland areas of Gyeonggi, Gyeongnam, and Gyeongbuk. The findings are consistent with those of a previous study showing that the observation frequency of these species was highest in Gyeongbuk [76].

Following the same approach employed for the mammals, we generated binary maps for each bird species utilizing the MTSS threshold from the MaxEnt results. We used these binary maps to identify common SAs for the four endangered birds. These SAs for endangered birds together covered approximately 22,356 km^2^, accounting for 22% of the total area of South Korea (100,413 km^2^) (Figure A2b). Unlike the results for the target mammals, the SAs for birds were distributed across Gyeonggi, Chungbuk, Chungnam, and Gyeongbuk, excluding forested regions. These findings suggest that inland areas where endangered birds have a high probability of occurrence were well-reflected in the distribution of the SAs.

**Figure 6:**
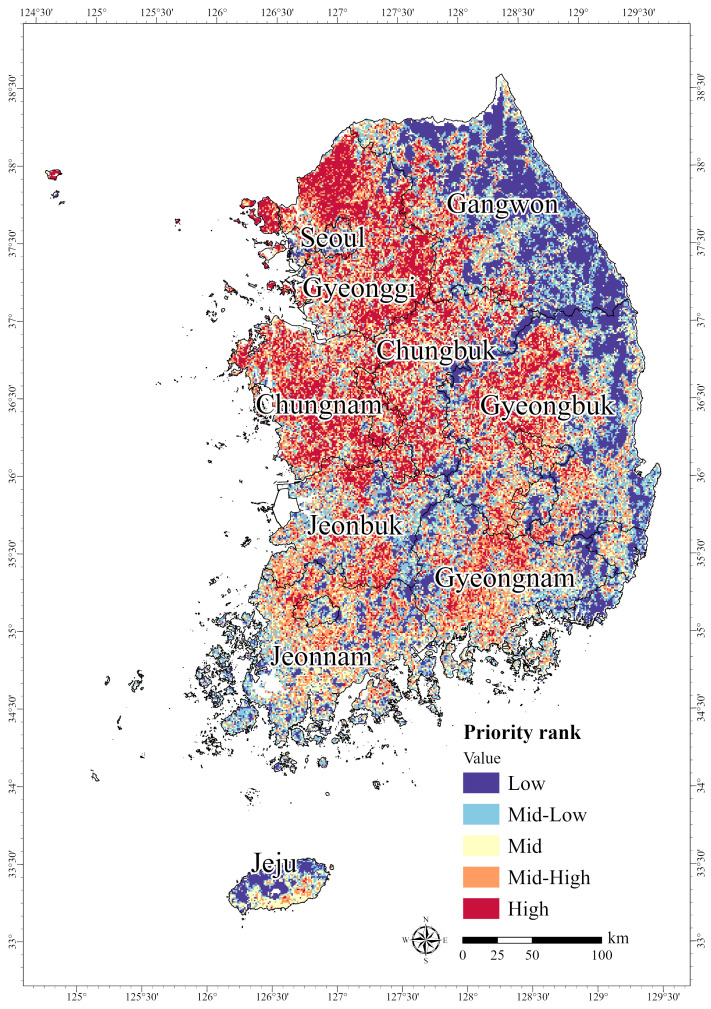
Prioritized areas of potential habitat for four birds designated as endangered in South Korea: *Falco subbuteo*, *Accipiter soloensis*, *Accipiter nisus*, and *Accipiter gentilis*.

To determine the PCAs for the endangered bird species, Zonation analysis was conducted by overlaying the species-specific distribution predictions from the MaxEnt model. We categorized the PCAs for endangered birds into five priority ranks and quantified the area for each ranking. Inland areas in the west and south of the Korean Peninsula, including Gyeonggi, Chungbuk, Chungnam, and Gyeongbuk, received the highest priority rank (Grade 5, “High”) (Figure 6), whereas certain areas in Gangwon and Gyeongbuk were assigned the lowest priority rank (Grade 1, “Low”). We examined the distribution of priority ranks across the nine administrative regions. As a result, the HPAs for endangered birds were most extensive in Gyeonggi (5056 km^2^), followed by Chungnam (3785 km^2^) and Gyeongbuk (3420 km^2^) (Table 5). The Grade 1 (“Low”) PCAs covered 18,845 km^2^; Grade 2 (“Mid-Low”) covered 19,214 km^2^; Grade 3 (“Mid”) covered 19,669 km^2^; Grade 4 (“Mid-High”) covered 19,220 km^2^; and Grade 5 (“High”) covered 19,597 km^2^ (Table 5). Unlike terrestrial fauna, the occurrence data for migratory birds, which are highly vagile species, may represent simple observation records that do not sufficiently reflect spatio-temporal factors [10,79]. Therefore, potential habitats for migratory birds may substantially differ from areas crucial for their survival in nature. Based on these findings, habitat conservation efforts targeting endangered birds need to prioritize HPAs but consider connectivity or key habitats for bird conservation while establishing conservation buffers.

### 3.3. Distribution of Endangered Amphibian and Reptile Species

This study analyzed the potential habitats for the three endangered amphibians *K. borealis*, *P. chosenicus*, and *D. suweonensis* and the three endangered reptiles *E. schrenckii*, *E. argus*, and *M. reevesii* using the MaxEnt model. The AUC ranged from a minimum of 0.7 to a maximum of 0.9, representing a high predictive model performance (Table 6). The AUC was 0.962 for *D. suweonensis*, 0.956 for *P. chosenicus*, and 0.856 for *K. borealis*, also indicating high model accuracy. Regarding the three reptiles, the AUC was 0.968 for *E. argus*, 0.778 for *M. reevesii*, and 0.724 for *E. schrenckii*. These results are supported by previous studies, which reported AUC values of 0.8–0.9 for amphibians and approximately 0.7 for reptiles in predicting distributions of endangered species [80].

We verified the contribution of the environmental variables used in the MaxEnt model to the distribution predictions of the endangered amphibians and reptiles. Elevation (DEM) was responsible for the highest contribution in the models for *K. borealis*, *P. chosenicus*, and *E. argus*, with these species demonstrating a higher probability of occurrence in low-altitude areas with a moderate slope (Table 6). The results align with previous research that identified elevation as a key environmental factor influencing the distribution of amphibians and reptiles [80]. For *E. schrenckii* and *M. reevesii*, land use and land cover (LULC) was the most influential factor associated with their occurrence, accounting for 23.5% and 18.3%, respectively, with a higher likelihood of occurrence in agricultural areas, grasslands, and wetlands. For *D. suweonensis*, temperature seasonality (Bio4) had the highest contribution, supported by related studies reporting a significant influence of temperature seasonality on the predicted distribution of this species [80].

**Figure 7:**
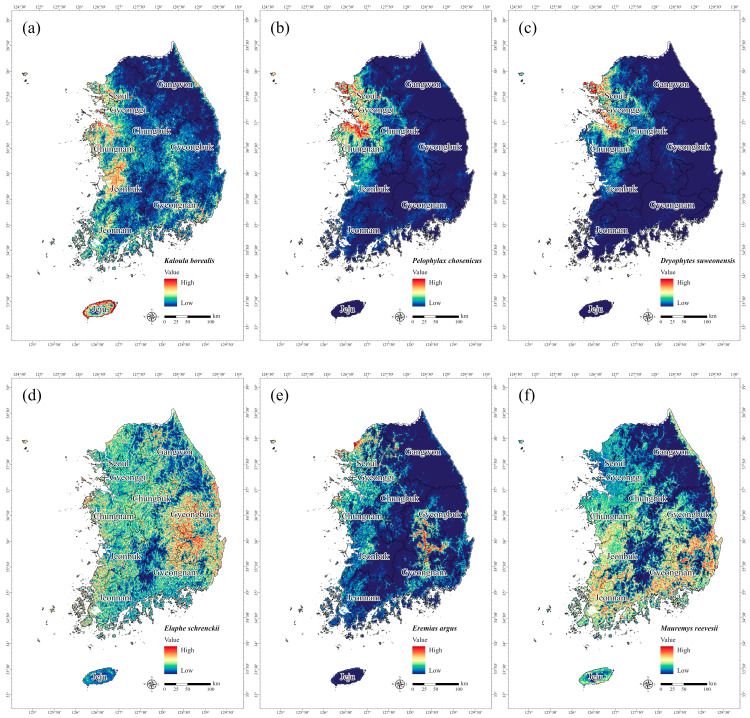
Potential distribution of three endangered amphibians designated in South Korea: (**a**) *Kaloula borealis*, (**b**) *Pelophylax chosenicus*, and (**c**) *Dryophytes suweonensis* and three endangered reptiles designated in South Korea: (**d**) *Elaphe schrenckii*, (**e**) *Eremias argus*, and (**f**) *Mauremys reevesii*. Red shading represents a high probability of species occurrence.

The potential habitats of the six endangered amphibian and reptile species were visualized as species distribution maps based on the MaxEnt analysis. We found that they predominantly occurred in lowland plains, agricultural areas, or areas near water bodies rather than in forested areas (Figure 7). In the case of endangered amphibians, the three species exhibited a high probability of predicted occurrence in the coastal areas of Gyeonggi and Chungnam. Notably, *K. borealis* was the only species of the six that exhibited high habitat suitability along the coastal regions on Jeju Island (Figure 7a). This finding was in accordance with previous studies that reported a high density of *K. borealis* on Jeju Island, where development pressure is relatively low [81]. For *P. chosenicus*, the known distribution includes Gyeonggi, Chungnam, Jeonnam, and Jeonbuk [82], and the model predictions in this study exhibited a similar pattern (Figure 7b). The populations of *D. suweonensis*, an endemic species of South Korea, were observed on some islands along the western coast [83], and the model predictions also indicated a high probability of occurrence in coastal areas and islands near Gyeonggi and Chungnam (Figure 7c).

The distribution range of *E. schrenckii* was the widest of the six target amphibians and reptiles, with particularly high predicted occurrence probabilities in Gyeongbuk and Gyeongnam (Figure 7d). Studies have reported that domestic populations of *E. schrenckii* are distributed nationwide, excluding Jeju Island [84]. In contrast, *E. argus* appeared to have a more localized distribution with a high probability of occurrence centered in certain areas of northern Gyeonggi and Gyeongbuk (Figure 7e). The findings conform to previous reports that *E. argus* was primarily found in the western coastal areas, including parts of Gyeongnam and Gyeongbuk [83]. For *M. reevesii*, previous studies have reported a high observation frequency in southern regions, excluding Jeju Island [85], with confirmed occurrences in Jeonbuk, Jeonnam, Gyeongbuk, and Gyeongnam [83]. Consistently, the MaxEnt model in the present study predicted a concentration of potential habitats along the coastal areas of Gyeongnam and Jeonnam, reflecting the habitat preferences of *M. reevesii* (Figure 7f).

Binary maps were also constructed for the endangered amphibians and reptiles. Overlay analysis of these binary maps indicated that the SAs for the endangered amphibians and reptiles accounted for approximately 4% (4009 km^2^) of the entire study area, the lowest proportion of the taxonomic groups. The SAs were primarily located in parts of Gyeonggi and Chungnam (Figure A2c). Endangered amphibians and reptiles had significantly fewer occurrence records than other taxa due to their limited habitat range and low population densities. These factors were reflected in the MaxEnt model, which is influenced by the number of species occurrence records, resulting in small SAs.

**Figure 8:**
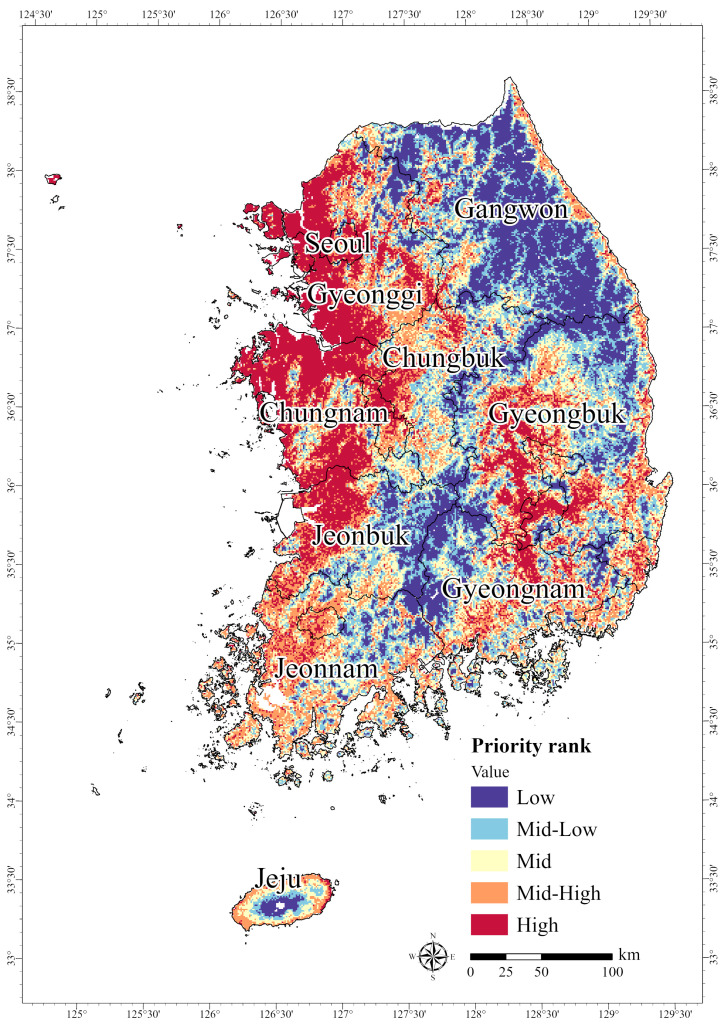
Prioritized areas of potential habitat for six amphibians and reptiles designated as endangered in South Korea: *Kaloula borealis*, *Pelophylax chosenicus*, *Dryophytes suweonensis*, *Elaphe schrenckii*, *Eremias argus*, and *Mauremys reevesii*.

Zonation analysis was also used to derive the PCAs for endangered amphibians and reptiles. HPAs with the highest priority rank (Grade 5, “High”) were found in coastal areas and water bodies in Gyeonggi and Chungnam, as well as parts of Gyeongbuk Province (Figure 8). In terms of the priority ranking within the nine administrative regions, Gyeonggi, with the highest ranking, contained the largest area at 5052 km^2^, followed by Chungnam at 5149 km^2^ and Gyeongbuk at 3212 km^2^ (Table 7). Overall, 18,823 km^2^ of the study area had a priority ranking of Grade 1 (“Low”); 19,199 km^2^ was Grade 2 (“Mid-Low”); 19,198 km^2^ was Grade 3 (“Mid”); 19,194 km^2^ was Grade 4 (“Mid-Low”); and 19,559 km^2^ was Grade 5 (“High”). Based on these results, to ensure the protection of habitats for endangered amphibians and reptiles, conservation strategies should be established for the HPAs in Gyeonggi and Chungnam Provinces.

### 3.4. Gap Analysis Results and Priority Conservation Areas

The Zonation analysis prioritized the potential habitats for endangered mammals, birds, amphibians, and reptiles by classifying them into five priority ranks. We derived the HPAs from the Zonation results and conducted a gap analysis with the first-grade areas from the ENM. The total area designated as first grade across South Korea is approximately 8595 km^2^, accounting for about 85.5% of the study sites. In addition, out of the total area of 16,855 km^2^ in Gangwon, 3406 km^2^ is designated as first grade, representing 40% of the overall first-grade area. The primary factor influencing the designation of first-grade areas is their vegetation, which is assessed based on survey data from the current vegetation map and forest age class [86]. As a result, Gangwon, with its dense forest cover, contains the largest proportion of first-grade areas on the ENM.

**Figure A3:**
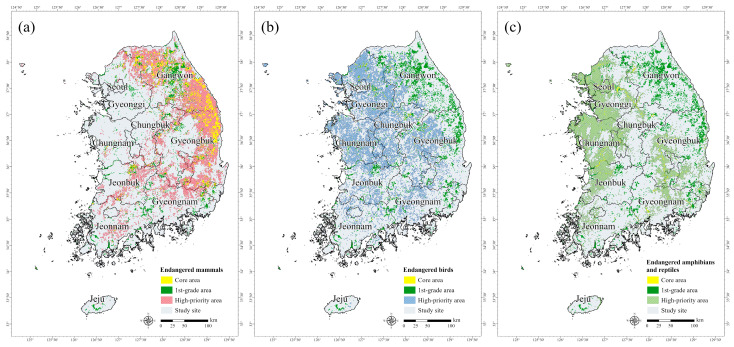
Overlap analysis for ecological areas and high-priority areas for the (**a**) five endangered mammals, (**b**) four endangered birds, and (**c**) six endangered amphibians and reptiles.

The priority conservation assessment of potential habitats for endangered species revealed that the HPAs classified as the highest priority (Grade 5, “High”) covered a larger area than the other priority ranks for all taxa. In particular, the HPAs for endangered mammals covered 19,590 km^2^, while those for endangered birds covered 19,597 km^2^ and those for endangered amphibians and reptiles covered 19,559 km^2^ (Table 8). In the gap analysis, the HPAs for each taxon were extracted and overlaid with the first-grade areas from the ENM, and the overlapping areas were defined as core areas of the PCAs (i.e., CPCAs) (Table 8). The analysis showed that the CPCAs for endangered mammals had the largest overlap, covering 3542 km^2^, which accounted for approximately 41.2% of the first-grade areas on the ENM (Table 8, Figure A3a). For endangered birds, the CPCAs covered 511 km^2^, with an overlap of 5.9% (Table 8, Figure A3b). Similarly, the CPCAs for endangered amphibians and reptiles covered 549 km^2^, representing an overlap of 6.3% (Table 8, Figure A3c). The overlap between the HPAs and the first-grade areas from the ENM was relatively low for endangered birds, amphibians, and reptiles compared to that for mammals. This confirmed that the areas with high potential for endangered birds, amphibians, and reptiles were rarely included in PAs recognized as ecologically valuable. However, areas with high suitability do not always necessarily coincide with areas of high conservation value. Dynamic threats can alter environmental conditions, affecting species distribution and biodiversity [87]. SDMs, including the MaxEnt model, effectively predict potential species range, but there are limitations in capturing the complex ecological interactions within landscapes [88]. Accordingly, the results suggest the importance of considering areas with a low overlapping ratio of the ENM as potential conservation targets of conservation strategies.

**Figure 9:**
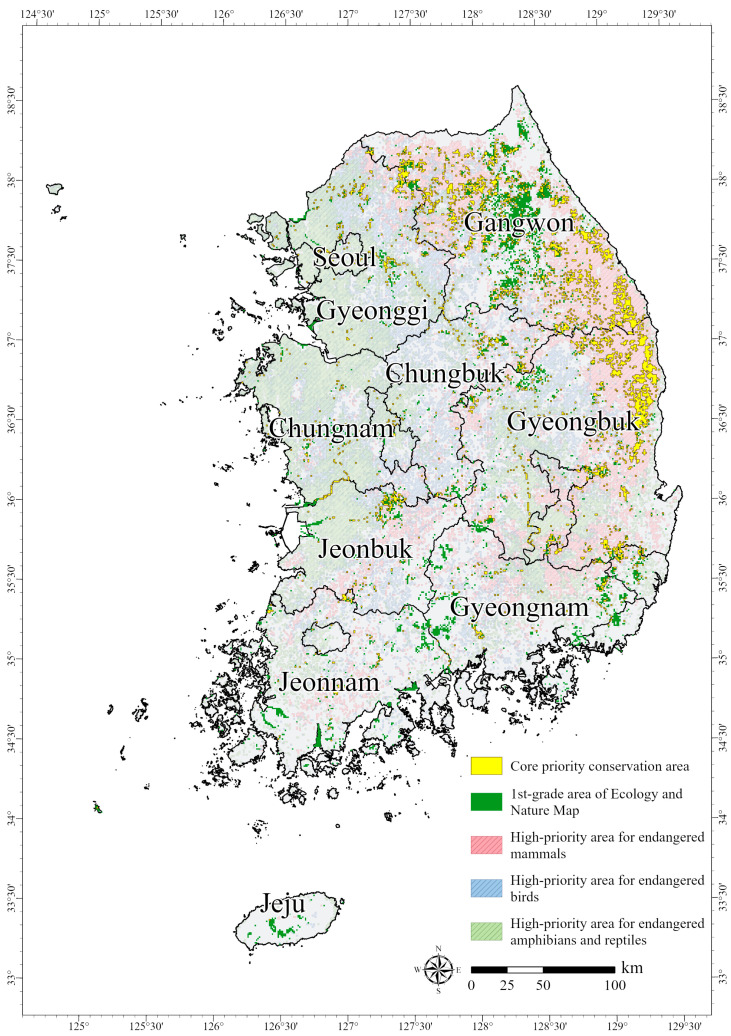
Priority conservation areas for endangered species in South Korea based on gap analysis results.

By synthesizing the results for all taxonomic groups, we derived CPCAs for endangered species located within the first-grade areas on the ENM. A total area covering approximately 4334 km^2^ was classified as CPCAs for protecting the habitats of endangered mammals, birds, amphibians, and reptiles (Figure 9). These CPCAs accounted for 50% of the first-grade areas on the ENM and 4.3% of the total area of South Korea. However, as shown in the gap analysis, the CPCAs for endangered mammals accounted for the largest proportion of the total area for CPCAs for all taxa. The CPCAs for endangered mammals were located mainly in Gangwon, which has a high concentration of first-grade areas on the ENM. In contrast, the CPCAs for endangered birds, amphibians, and reptiles accounted for a relatively small proportion of the overall results.

The gap analysis indicated that the discrepancy between endangered mammals and the other taxonomic groups was primarily due to differences in the spatial distribution patterns of the SAs and the PCAs for each group. Endangered mammals predominantly inhabit forested environments, leading to the presence of core habitats in Gangwon and Gyeongbuk on the eastern Korean Peninsula. Due to their habitat preferences, the overlap between the PCAs and the first-grade areas on the ENM, where vegetation characteristics are key evaluation criteria, was the highest in Gangwon for mammals. In contrast, the potential habitats for endangered birds, amphibians, and reptiles were more commonly found in lowlands or along coastal regions rather than in forested areas. As a result, the overlap between their PCAs and the first-grade areas on the ENM was significantly lower than that for endangered mammals. In addition, field surveys can detect mammals relatively easily via visible traces such as scat, footprints, and feces. For birds, however, different habitat use patterns between breeding and non-breeding seasons and field surveys that rely on vocalization as a presence indicator [77] make it difficult to obtain accurate information on endangered populations. In addition, protecting nest sites has been identified as an effective strategy for increasing survival rates and supporting bird population conservation [89,90,91]. Therefore, surveys of nesting areas are essential for informing targeted management efforts. For amphibians and reptiles, research on their basic ecology has only recently gained traction in South Korea, and the habitat use of amphibians and reptiles varies depending on breeding season and environmental factors; thus, it is difficult to estimate their population status accurately [84,92].

To effectively conserve endangered species in South Korea, comprehensive assessments and research that consider their ecology and distribution patterns are required. Amphibians, in particular, hold significant ecological importance because they are highly sensitive to climate change. For example, research on the habitat of *Dryophytes suwonensis*, a species endemic to South Korea, has significant academic value [93]. Furthermore, given the high development pressure in low-altitude areas inhabited by amphibians and reptiles, further research on their core habitats is essential for appropriate conservation [80]. Amphibians and reptiles are also the primary prey of many vertebrates such as mammals and birds [94,95]; thus, understanding their ecological roles can provide a foundation for broader ecological analysis across taxonomic groups.

In addition, to ensure the development of effective conservation strategies, it is necessary to introduce evaluation criteria that adequately account for the potential habitats of endangered birds, amphibians, and reptiles, which are not primarily associated with forested areas. Additional research on the basic ecology and distribution of diverse taxonomic groups would allow them to be integrated into the evaluation process for different grades of the ENM. Consequently, these efforts would contribute to the establishment of more effective conservation strategies that encompass all endangered species in South Korea. A refined assessment of grading zones within the ENM could also provide a basis for expanding the first-grade areas or improving the evaluation criteria. This strategic approach would enhance biodiversity conservation and management by ensuring more effective, data-driven conservation planning.

## 4. Conclusions

Identifying PAs that include the potential habitats of endangered species is essential for their effective conservation. This study employed the MaxEnt model and Zonation analysis to identify priority areas for the conservation of endangered species and conducted a gap analysis to examine the overlap between PCAs and the ENM. However, the same set of environmental variables was applied to all of the target species in the MaxEnt model to simplify the input data for computational efficiency. This approach may not completely reflect the complete ecological characteristics of individual species. The environmental factors of habitats vary by taxon, which is important for the conservation of species in nature. Since the environmental factors are often only indirectly related to the area where species are found to occur, this approach may not completely reflect the ecological characteristics of individual species. Further analysis that categorizes environmental variables by species or taxonomic group is thus required to improve the accuracy of the modeling results.

Another limitation of this study is that the occurrence data for endangered amphibians and reptiles were significantly less abundant than those for mammals and birds, and the restricted habitat ranges of certain species led to a regionally biased distribution in the model predictions. With the fifth NNES (2019–2023) currently being completed and the data gradually being released, future research integrating more refined spatial datasets for endangered species and their habitats is expected to enhance the accuracy of the proposed model.

This study provides the predicted distribution data for endangered species inhabiting South Korea by analyzing habitat suitability and conservation priorities for different taxonomic groups. Furthermore, by conducting an overlap analysis with the first-grade areas on the ENM, the study highlights areas requiring additional conservation efforts. The study results highlight the need for more field surveys focusing on endangered birds, amphibians, and reptiles, as well as the importance of integrating unprotected potential habitats into conservation strategies.

Future studies incorporating comparative analysis with alternative models or the validation of results through combined modeling approaches may facilitate the development of optimized species distribution models. The findings of this study are expected to serve as fundamental reference data for environmental policy and conservation planning. Specifically, the identified priority areas can guide land-use planning and environmental impact assessments by highlighting critical areas and providing mitigation strategies prior to project implementation. Furthermore, the results may assist in developing management plans that enhance national ecological networks and improve habitat connectivity. The findings of this study can provide a scientific basis for the designation and management of PAs and continued research on conservation strategies for endangered species habitats.

## Figures and Tables

**Table 1:** Information on 15 endangered species and their occurrence data used in the MaxEnt model (total of 6166 species occurrence points).

Taxon	Species	IUCN Criteria	MOE Class	Survey Records	Occurrence Points
Mammals	*Prionailurus bengalensis*	LC	II	4410	2374
	*Lutra lutra*	NT	I	2401	1481
	*Martes flavigula*	LC	II	755	446
	*Pteromys volans*	LC	II	100	85
	*Naemorhedus caudatus*	VU	I	128	51
Total				7794	4437
Birds	*Falco subbuteo*	LC	II	738	488
	*Accipiter soloensis*	LC	II	626	466
	*Accipiter nisus*	LC	II	337	284
	*Accipiter gentilis*	LC	II	262	214
Total				1963	1452
Amphibians	*Kaloula borealis*	LC	II	109	88
	*Pelophylax chosenicus*	VU	II	80	60
	*Dryophytes suweonensis*	EN	I	30	26
Total				219	174
Reptiles	*Elaphe schrenckii*	LC	II	82	63
	*Eremias argus*	LC	II	33	25
	*Mauremys reevesii*	EN	II	18	15
Total				133	103

**Table 2:** MaxEnt model results for five endangered mammal species.

Taxon	Species	Test AUC	Logistic Threshold (MTSS)	Variable Contribution (%)
Mammals	*Naemorhedus caudatus*	0.964	0.389	Precipitation in driest quarter (32.3%) > elevation (11.8%) > forest age classes (10.9%) > topographic wetness index (7.7%) > gradient (7.5%)
	*Martes flavigula*	0.823	0.434	Elevation (42.9%) > isothermality (13.6%) > gradient (8.9%) > temperature seasonality (6.7%) > precipitation in driest quarter (6.6%)
	*Pteromys volans*	0.793	0.393	Elevation (18.7%) > precipitation in driest quarter (15.1%) > land use and land cover (8.7%) > gradient (8.3%) > forest age classes (8.1%)
	*Lutra lutra*	0.743	0.445	Distance from water (33.8%) > temperature seasonality (10.7%) > elevation (10.2%) > precipitation in driest quarter (8.1%) > precipitation in wettest quarter (7.7%)
	*Prionailurus bengalensis*	0.658	0.478	Temperature seasonality (38.3%) > normalized difference vegetation index (9.5%) > gradient (9.0%) > isothermality (8.4%) > land use and land cover (7.3%)

**Table 3:** Priority conservation areas for five endangered mammals by administrative regions (unit: km^2^).

Province	Priority Rank				
Classification	1 (Low)	2 (Mid-Low)	3 (Mid)	4 (Mid-High)	5 (High)
Gangwon	562	3989	1484	3163	7218
Gyeongbuk	1924	2547	3536	4641	6973
Gyeongnam	1343	1457	4054	3294	1419
Jeonbuk	1251	1441	2010	2018	1145
Jeonnam	1258	2256	4189	2504	898
Gyeonggi	6540	2794	550	614	872
Chungbuk	1082	2285	1373	1872	793
Chungnam	3379	2223	1999	1075	263
Jeju	1482	219	16	33	9
Total	18,821	19,211	19,211	19,214	19,590

**Table 4:** MaxEnt model results for four endangered bird species.

Taxon	Species	Test AUC	Logistic Threshold (MTSS)	Variable Contribution (%)
Birds	*Accipiter soloensis*	0.755	0.471	Elevation (41.8%) > precipitation in driest quarter (8.0%) > precipitation in wettest quarter (6.4%) > temperature seasonality (6.0%) > gradient (4.7%)
	*Accipiter gentilis*	0.738	0.461	Temperature seasonality (21.5%) > precipitation in wettest quarter (10.0%) > distance from road (9.7%) > normalized difference vegetation index (8.1%) > land use and land cover (0.7%)
	*Falco subbuteo*	0.725	0.473	Elevation (14.9%) > land use and land cover (11.0%) > isothermality (10.0%) > distance from water (8.8%) > forest age classes (8.0%)
	*Accipiter nisus*	0.716	0.468	Distance from water (14.9%) > land use and land cover (11.0%) > elevation (10.6%) > distance from agricultural area (9.2%) > temperature seasonality (8.5%)

**Table 5:** Priority conservation areas for four endangered birds by administrative regions (unit: km^2^).

Province	Priority Rank				
Classification	1 (Low)	2 (Mid-Low)	3 (Mid)	4 (Mid-High)	5 (High)
Gyeonggi	590	1206	1983	2563	5056
Chungnam	187	1000	1713	2256	3785
Gyeongbuk	4292	4235	3830	3848	3420
Chungbuk	807	1196	1549	1485	2365
Gangwon	7250	3682	2412	1605	1464
Jeonbuk	958	1924	1663	1893	1426
Gyeongnam	2190	2950	2459	2743	1236
Jeonnam	1798	2745	3504	2676	827
Jeju	773	276	556	151	18
Total	18,845	19,214	19,669	19,220	19,597

**Table 6:** MaxEnt model results for six endangered amphibian and reptile species.

Taxon	Species	Test AUC	Logistic Threshold (MTSS)	Variable Contribution (%)
Amphibians	*Dryophytes suweonensis*	0.962	0.503	Temperature seasonality (32.5%) > elevation (32.0%) > isothermality (9.0%) > land use and land cover (6.1%) > distance from agricultural area (3.4%)
	*Pelophylax chosenicus*	0.956	0.345	Elevation (42.9%) > temperature seasonality (18.0%) > isothermality (16.3%) > land use and land cover (4.9%) > precipitation in driest quarter (3.9%)
	*Kaloula borealis*	0.856	0.391	Elevation (35.1%) > land use and land cover (13.9%) > precipitation in driest quarter (11.3%) > gradient (6.8%) > forest age classes (6.2%)
Reptiles	*Eremias argus*	0.968	0.491	Elevation (25.6%) > temperature seasonality (18.9%) > land use and land cover (14.2%) > precipitation in driest quarter (9.0%) > forest age classes (6.4%)
	*Mauremys reevesii*	0.778	0.398	Land use and land cover (18.3%) > elevation (14.1%) > normalized difference vegetation index (9.7%) > gradient (9.4%) > temperature seasonality (8.4%)
	*Elaphe schrenckii*	0.724	0.440	Land use and land cover (23.5%) > elevation (12.8%) > precipitation in driest quarter (12.2%) > aspect (8.9%) > distance from water (7.4%)

**Table 7:** Priority conservation areas for six endangered amphibians and reptiles by administrative regions (unit: km^2^).

Province	Priority Rank				
Classification	1 (Low)	2 (Mid-Low)	3 (Mid)	4 (Mid-High)	5 (High)
Gyeonggi	820	1040	1481	2521	5502
Chungnam	148	676	1281	1678	5149
Gyeongbuk	3315	4975	4605	3515	3212
Jeonbuk	1524	1678	1342	1345	1969
Gyeongnam	2150	2440	2934	2677	1360
Jeonnam	1083	2064	2870	4027	1032
Chungbuk	1103	1809	1973	1645	872
Gangwon	8406	4167	2168	1267	387
Jeju	274	350	544	519	76
Total	18,823	19,199	19,198	19,194	19,559

**Table 8:** Proportion of high-priority conservation areas for endangered species by taxon based on gap analysis (unit: km^2^).

Classification	Mammals	Birds	Amphibians and Reptiles
High-priority areas (HPAs)	19,590	19,597	19,559
Priority conservation areas (PCAs)	3542 (41.2%)	511 (5.9%)	549 (6.3%)
Total areas	4334 (50.4%)		

## Data Availability

The data that support the findings of this study are available from the corresponding author upon reasonable request.

## Appendix A

**Table A1:** Ecological features of 15 endangered species in South Korea classified according to the IUCN Red List of Threatened Species (Least Concern [LC], Near Threatened [NT], Vulnerable [VU], Endangered [EN]) and Endangered Wildlife Classes I and II designated by the Korea Ministry of Environment (MOE).

Class	Species Name		IUCN Criteria	MOE Class	Species Ecology
	Common Name	Scientific Name			
Mammalia	Leopard cat	*Prionailurus bengalensis*	LC	II	Widely distributed across various habitat types, including forests, agricultural areas, and riparian zones Functions as a top predator in South Korea, where large carnivores have disappeared
	Eurasian otter	*Lutra lutra*	NT	I	Primarily inhabits aquatic environments, including inland rivers, streams, and coastal areas Acts as a bioindicator of aquatic ecosystem health
	Yellow-throated marten	*Martes flavigula*	LC	II	Prefers forest environments with complex structures consisting of broadleaf forests or mixed forests Acts as a top predator and bioindicator for forest ecosystem health
	Siberian Flying squirrel	*Pteromys volans*	LC	II	Has high population densities in broadleaf and mixed forests, nesting in tree cavities Acts as an indicator species for assessing forest ecosystem health
	Long-tailed goral	*Naemorhedus caudatus*	VU	I	Primarily inhabits rocky cliffs and mountainous terrain with high elevations and steep slopes Populations have declined due to habitat loss from development and extreme snowfall
Aves	Eurasian hobby	*Falco subbuteo*	LC	II	Widely utilizes forested environments near agricultural areas and wetlands during the summer Populations have declined due to a loss of habitat and food sources driven by land-use changes
	Chinese sparrowhawk	*Accipiter soloensis*	LC	II	Distributed nationwide, primarily inhabiting forests, plains, and agricultural areas during the summer Populations have declined due to urbanization and lower food availability
	Eurasian sparrowhawk	*Accipiter nisus*	LC	II	Inhabits forests, plains, and urban areas during the winter Requires international conservation efforts due to habitat loss and a reduction in food sources caused by development
	Northern goshawk	*Accipiter gentilis*	LC	II	Uses environments such as forests, agricultural areas, rivers, and urban areas during winter Populations have declined due to habitat loss and a decrease in food sources
Amphibia	Narrow-mouthed toad	*Kaloula borealis*	LC	II	Exhibits high population densities in wetlands and agricultural areas Decline in habitat range due to habitat destruction and pesticide use
	Korean golden frog	*Pelophylax chosenicus*	VU	II	Prefers agricultural areas as its primary habitat Highly sensitive to human activity, including urbanization and development
	Suweon tree frog	*Dryophytes suweonensis*	EN	I	Primarily distributed in plains, wetlands, and agricultural areas Endemic species with a small and fragmented population
Reptilia	Rat snake	*Elaphe schrenckii*	LC	II	Found in forests, streams, and agricultural areas Population decline due to habitat destruction, habitat loss, and poaching
	Korean tiger lizard	*Eremias argus*	LC	II	Primarily distributed in coastal areas Functions as an indicator species for assessing the health of both marine and terrestrial ecosystems
	Reeve’s turtle	*Mauremys reevesii*	EN	II	Mostly inhabits grasslands, plains, and agricultural areas Experiencing rapid population decline due to habitat destruction, overexploitation, and competition with invasive species

**Table A2:** Classification of forest age classes in the 1:5000 forest map.

Code	Description
1	1–10-year-old tree canopy occupation ratio of 50% or more
2	11–20-year-old tree canopy occupation ratio of 50% or more
3	21–30-year-old tree canopy occupation ratio of 50% or more
4	31–40-year-old tree canopy occupation ratio of 50% or more
5	41–50-year-old tree canopy occupation ratio of 50% or more
6	51–60-year-old tree canopy occupation ratio of 50% or more
7	61–70-year-old tree canopy occupation ratio of 50% or more
8	71–80-year-old tree canopy occupation ratio of 50% or more
9	81–90-year-old tree canopy occupation ratio of 50% or more

**Table A3:** Summary of the 16 environmental variables used in the MaxEnt model.

Classification	Variable	Description	Data Type	Data Source
Topography	DEM	Elevation	Continuous	National Geographic Information Institute (2014) (https://map.ngii.go.kr, accessed on 11 October 2023)
	Slope	Gradient		
	Aspect	Aspect		
	TWI	Topographic wetness index		Korea Institute of Geoscience and Mineral Resources (2020) (https://www.bigdata-environment.kr, accessed on 20 June 2024)
Distance	Resid	Distance from residential area		Environmental Geographic Information Service (2023) (https://egis.me.go.kr, accessed on 9 May 2024)
	Water	Distance from water		
	Used	Distance from used area		
	Agri	Distance from agricultural area		
	Road	Distance from road		
Climate	Bio3	Isothermality (Bio2)/(Bio7)		Worldclim (1970–2000) (https://worldclim.org, accessed on 20 June 2024)
	Bio4	Temperature seasonality (standard deviation ×100)		
	Bio16	Precipitation in wettest quarter		
	Bio17	Precipitation in driest quarter		
Vegetation	NDVI	Normalized difference vegetation index		Korea Institute of Geoscience and Mineral Resources (2022) (https://www.bigdata-environment.kr, accessed on 20 June 2024)
	AGCLS	Forest age class	Categorical	Korea Forest Service (2023) (https://map.forest.go.kr, accessed on 21 September 2023)
Land cover	LULC	Land use and land cover		Environmental Geographic Information Service (2023) (https://egis.me.go.kr, accessed on 9 May 2024)
